# VPO1 mediates oxidation of LDL and formation of foam cells

**DOI:** 10.18632/oncotarget.9193

**Published:** 2016-05-05

**Authors:** Youfeng Yang, Ruizheng Shi, Zehong Cao, Guogang Zhang, Guangjie Cheng

**Affiliations:** ^1^ Division of Pulmonary, Allergy and Critical Care Medicine, Department of Medicine, University of Alabama at Birmingham, Birmingham, AL, USA; ^2^ Department of Cardiovascular Medicine, Xiangya Hospital, Central South University, Changsha, China

**Keywords:** heme-containing peroxidase, free radical, low-density lipoprotein, oxidized lipids, foam cells, Gerotarget

## Abstract

Deposition of oxidized-LDL in vascular walls is essential in the initiation of atherosclerosis. Oxidation of LDL has been attributed to myeloperoxidase as its generation of potent oxidants. However, the exact mechanism of LDL oxidation and foam cell formation in atherosclerosis remains to be elucidated. Vascular peroxidase-1 (VPO1), a newly-identified heme-containing peroxidase, is primarily expressed in cardiovascular systems, and secreted into the circulation. The present study evaluates VPO1-mediated LDL oxidation and its role in atherosclerosis. VPO1 was first demonstrated binding to LDL. VPO1-mediated oxidation of proteins and lipids in LDL was verified by a variety of methods including immunoblot analysis, free tryptophan assay, UV absorbance, and thiobarbituric acid assay. VPO1-oxidized LDL caused accumulation of LDL in monocyte-like cells and promoted formation of foam cells. Administration of inflammation factors, LPS or TNF-α, induced increasing expression of VPO1 in aorta and secretion to plasma. TNF-α also promoted formation and retention of VPO1-oxidized LDL in aortic walls. Our data suggest that VPO1 contributes to oxidation and retention of LDL in vessel walls, and formation foam cells, indicating VPO1 as a novel potential mediator of atherosclerosis.

## INTRODUCTION

Atherosclerosis is a multistep, multifactorial and inflammatory disease, characterized by deposition of lipoprotein, monocyte recruitment, and formation of foam cells in arterial walls [1–3]. The pathogenesis of atherosclerosis includes initiation and lesion progress. Oxidation of low-density lipoprotein (LDL) plays an important role both in lesion initiation and progression. Oxidation of LDL may occur in plasma, tissues, as well as the arterial intima [4, 5]; oxidation of LDL in arterial intima promotes formation of foam cells and retention of LDL in vessel walls, as well as induces expression of inflammatory factors [6]. Oxidized LDL (oxLDL) in arterial walls is considered as one of the major onset mechanisms for the pathogenesis of atherosclerosis [7].

A variety of enzymes are involved in the oxidation of LDL. Of which, leukocyte-derived myeloperoxidase (MPO) has been extensively studied [8, 9]. MPO is highly expressed in neutrophils, in which MPO counts as approximately 5% of the dry weight [10]. Physiologically, MPO plays an important role in the host defense by killing invading microbes through generation of HOCl in the presence of H_2_O_2_ and Cl^−^. Under pathological conditions, MPO is thought as a major mediator for oxidation of LDL since MPO generates multiple oxidants to convert LDL to an atherogenic form *in vitro* [3, 11, 12]. However, MPO is strictly expressed in neutrophils and monocytes; small amount of secreted MPO may indirectly enter vessel walls by transcytosis during acute inflammation [13]. Lusis and colleagues reported that unlike humans, mouse monocytes have little MPO, while atherosclerotic lesions in MPO-deficient mice are about 50% larger than wild-type control [14]. MPO-transgenic mice, whose macrophages expressed human MPO, revealed more severe lesion of atherosclerosis [15]. These data imply a role for another (non-MPO) heme-containing peroxidase (hPx) in arterial walls in the pathogenesis of atherosclerosis.

Our laboratory recently identified and characterized a novel member of hPx family, named vascular peroxidase-1 (VPO1) [16, 17]. It is the mammalian homologue of Drosophila peroxidasin (PXDN) [18]. Unlike MPO, VPO1 is highly expressed in vascular endothelial cells (VECs), vascular smooth muscle cells (VSMCs), heart and lung; VPO1 proteins are also secreted into the circulation [16]. VPO1 mediates generation of HOCl and is the second mammalian hPx generating HOCl [19]. Herein, we explore the pathological role of VPO1 in atherogenesis.

## RESULTS

### VPO1 binds to LDL

**Figure 1:**
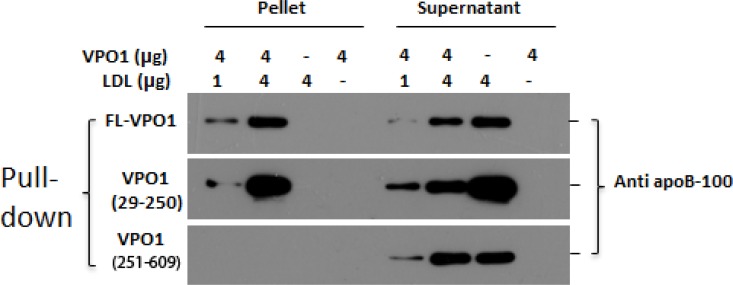
VPO1 binds to LDL Full-length rVPO1, rVPO1 (29-250aa) or rVPO1 (251-609aa) was incubated with LDL as described in Materials and Methods. The recombinant proteins were pulled down by HisPur^®^ Cobalt resin. The control groups only contained rVPO1 or LDL alone. The pallets and supernatants were collected, respectively. The pellets and supernatants were subject to immunoblot analysis. apoB-100 in LDL was detected by using anti-apoB-100 antibody and visualized by chemiluminescence. The data are representative of two independent experiments.

VPO1 is unique in hPx family; it contains five leucine-rich repeats (LRRs) and four immunoglobulin (Ig) C2 domains at the N-terminus [17]. These structures are proposed for protein and protein interaction [17]. In addition, VPO1 expresses in vessel walls and is secreted into the circulation [16]. Based on its properties of biochemistry and cell biology, we asked if VPO1 interacts with LDL. Full-length and truncated VPO1 were incubated with LDL. The pull-down experiments showed that full-length VPO1 and VPO1 29-250 aa (containing LRRs), not VPO1 251-609aa (containing Ig C2 domains), bound to LDL. The interaction was at dose-dependent manner (Figure 1). The data strongly support that VPO1 interacts with LDL.

### VPO1 mediates LDL oxidation

**Figure 2:**
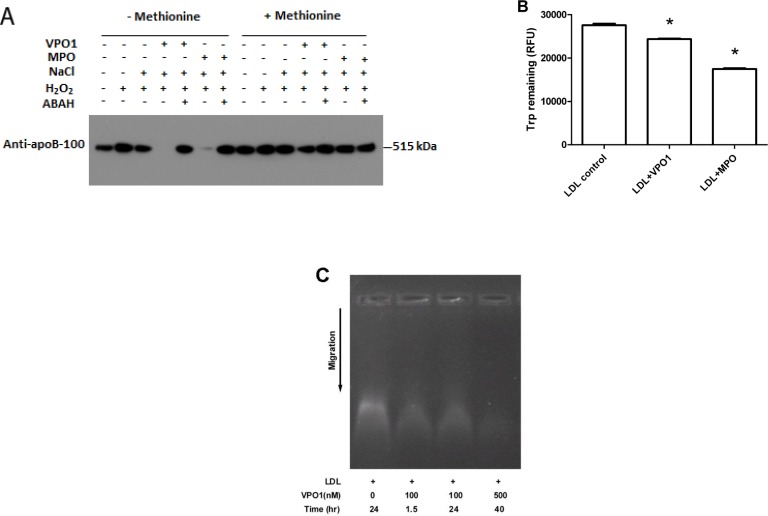
VPO1 mediates protein oxidation of LDL **A.** apoB-100 was oxidized by VPO1. LDL was incubated in VPO1/H_2_O_2_/Cl^−^ or MPO/ H_2_O_2_/Cl^−^ (positive control) as described in Materials and Methods. The oxidized samples were subject to immunoblot analysis using anti-apoB-100 antibody. In some groups, methionine was added at 5 mmol/L while ABAH at 1 mmol/L. **B.** Tryptophan residues in LDL were oxidized by VPO1 and MPO. LDL was incubated as in A. The contents of tryptophan in LDL were determined. *p < 0.05 *vs*. LDL control. *n* = 3. **C.** Agarose gel electrophoresis of VPO1-oxidized LDL. LDL was oxidized by VPO1 as described in A. Native LDL and VPO1-oxidized LDL were analyzed by electrophoresis on 0.8% agarose gel and visualized by staining with Nile red.

MPO plays an important role in atherosclerosis via oxidation of proteins and lipids [3, 11, 12]. MPO oxidizes proteins and lipids primarily through generation of HOCl, a potent oxidant [20]. apoB-100 is the protein component of LDL and readily oxidized by HOCl to form dimers and high-molecular-mass aggregates [21]. VPO1 was reported as the second mammalian enzyme generating HOCl [19]. To assess if VPO1 mediates oxidation of LDL, LDL was incubated with VPO1, H_2_O_2_ and Cl^−^. The product was subject to immunoblot analysis using the anti-apoB antibody. As shown in Figure 2A, the VPO1/H_2_O_2_/Cl^−^ system catalyzed apoB-100 oxidation, revealing loss of apoB-100 band. It is assumed that oxidation of apoB-100 caused apoB-100 degradation and/or conformation changes of the recognition site of the apoB-100 antibody. Oxidation pattern of apoB-100 by VPO1 was similar to that of MPO (Figure 2A). The VPO1-mediated oxidation was inhibited by methionine (a scavenger of HOCl) and ABAH (peroxidase inhibitor) (Figure 2A). These data suggest that VPO1 is able to oxidize apoB-100 in LDL *via* generation of HOCl.

To further evaluate the oxidation of proteins, free tryptophan residues in LDL were measured. Significant loss of free tryptophan residues was detected in VPO1-mediated reactions, similar to that of MPO (Figure 2B). However, VPO1 showed weaker effect on oxidizing tryptophan residues than that of MPO, consistent with weaker enzymatic activity of VPO1 [17, 22]. The relative electrophoretic mobility of LDL oxidation was also evaluated by using agarose gel electrophoresis. The LDL migration distance was increased in VPO1-mediated group (Figure 2C), where the ratios of the relative mobility of native LDL, 100 nmol/L VPO1-mediated oxLDL (1.5 hrs and 24 hrs) and 500 nmol/L VPO1-mediated oxLDL were 0.6, 0.64, 0.64 and 0.7. In addition, the increased relative electrophoretic mobility was a function of the concentration of VPO1 and the incubation time (Figure 2C).

**Figure 3:**
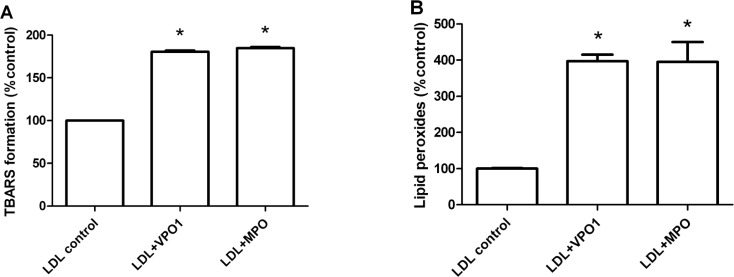
VPO1 mediates lipid oxidation of LDL LDL was oxidized by VPO1 or MPO as described in Figure 2A. Oxidized lipids in LDL were determined by thiobarbituric acid assay (**A.)** and lipid peroxides assay (**B.)** Native LDL was as control. *p < 0.05 *vs*. LDL control. *n* = 3.

We further evaluated VPO1-mediated lipid oxidation. An elevated concentration of thiobarbituric acid-reactive substances was detected in VPO1-mediated oxLDL, indicating accumulation of aldehydes from lipid oxidation (Figure 3A). In addition, UV absorbance at 265 nm was carried out to measure lipid peroxide products. Similar to MPO, VPO1-mediated lipid peroxide was significantly increased (Figure 3B). Taken together, our data strongly support that VPO1 mediates the broad oxidation of LDL.

### VPO1-oxidized LDL induces foam cell formation

**Figure 4:**
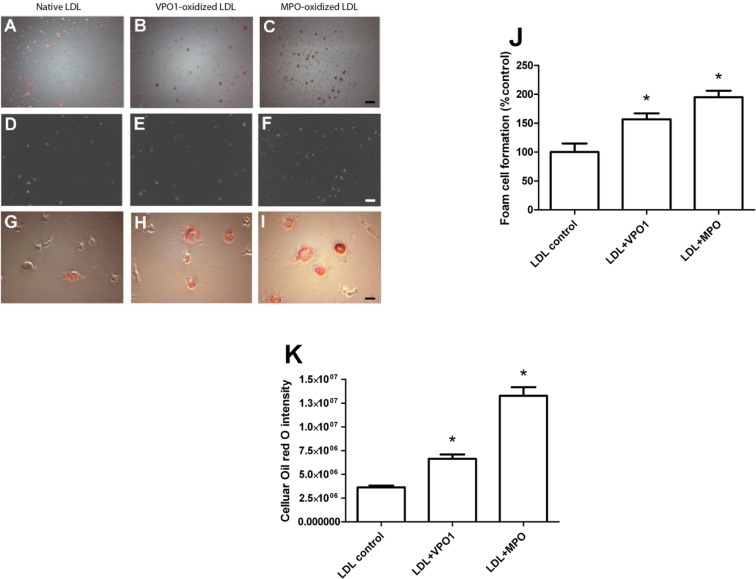
VPO1-oxidized LDL induces foam cell formation Human THP-1 monocytic cells were first differentiated into macrophages by PMA. The primed macrophages were incubated with native LDL, VPO1-oxidized LDL or MPO-oxidized LDL (100 μg/mL). Cells, which were placed on coverslips, were stained by Oil Red O. Images were recorded by microscope. **A.**-**C.** Oil Red O dye staining. Scale bar, 60 μm. Magnification, 100x. **D.**-**F.** Bright field images. Scale bar, 60 μm. Magnification, 100x. **G.**-**I.** Oil Red O dye staining. Scale bar, 15 μm. Magnification, 400x. **J**. Plot and statistical analysis of foam cell formation. **K**. CTD (corrected total red oil dye) is analysis by ImageJ software. *p < 0.05 *vs*. LDL control. *n* = 3.

Formation of foam cells is hallmark of atherosclerosis [2, 3, 23]. An array of evidence indicates that MPO promotes cholesterol retention in macrophage-rich human atherosclerotic tissues and stimulates foam cell formation [11]. Our group previously reported that VPO1 can impair plasma lipid clearance *via* oxidation of apoE [24], a major protein in VLDL. Herein, we further assessed VPO1-mediated foam cell formation *via* oxidation of LDL using human monocytic cells (THP-1). THP-1 cells were differentiated into microphages before the experiments of foam cell formation. Like MPO-oxidized LDL, VPO1-oxidized LDL caused lipid accumulation in macrophages (Figure 4A-4C and 4G-4I). The similar results were seen under bright field (Figure 4D-4F) as well as low (100x) and high (400x) magnification (Figure 4A-4F and 4G-4I). Increasing number of foam cells was observed in VPO1-oxidized LDL, similar to positive control of MPO-oxidized LDL (Figure 4J). Consistently, accumulated LDL levels inside the cells were elevated (Figure 4K).

### LPS and TNF-α induce VPO1 expression in aorta

**Figure 5:**
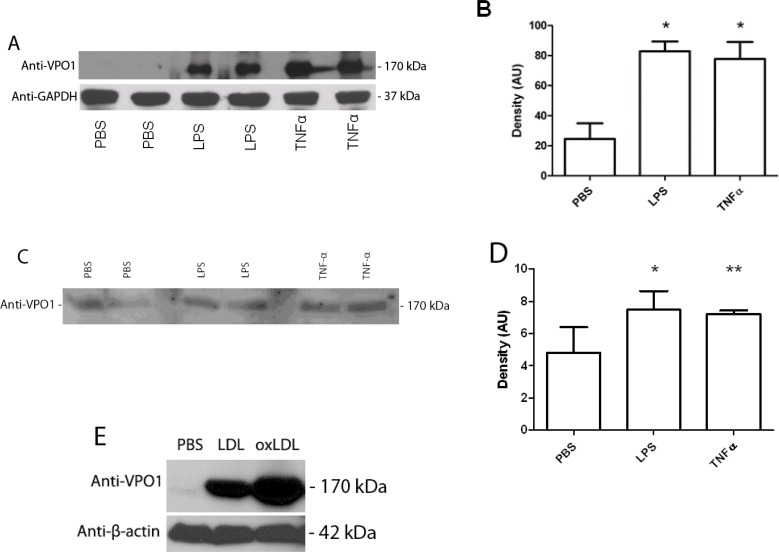
LPS, TNF-α and LDL induce VPO1 expression in aorta and secretion into plasma LPS (500 ng/g) or TNF-α (8 ng/g) was injected into mouse tail vein. PBS was as control. After 24 hrs, mouse aorta and plasma were harvested for immunoblot analysis. **A.** Immunoblot analysis of VPO1 expression in aorta. **B.** Densitometry of “A”. **C.** Immunoblot analysis of VPO1 expression in plasma. **D.** Densitometry of “C”. *p < 0.05 *vs*. PBS; *n* = 4. **E**. LDL induces VPO1 expression in aorta. 52.5 μg of LDL or oxLDL were injected into mouse tail vein (3 mice/group). PBS was as control. After 24 hrs, mouse aorta was harvested for immunoblot analysis. Representative blot was shown.

Atherosclerosis is a chronic inflammatory disease, in which inflammatory factors play a critical role in the pathogenesis of atherosclerosis not only initiating but also worsening the process [25]. It was reported that administration of LPS or TNF-α induced vascular inflammatory responses of mice. For example, LPS caused wide-spread vascular inflammatory responses, and worked as an aggravating factor in mouse apoE-deficient atherosclerosis [26, 27]. Elevated blood levels of TNF-α lead to inflammatory responses of VECs and VSMCs including abnormal interaction of vascular endothelial cell-blood cell [28]. VPO1 is expressed in a variety of cell types and tissues including VECs and VSMCs, and is secreted into the circulation. We previously reported that LPS and TNF-α induced VPO1 expression and secretion in cultured VECs [16]. We then further examined if LPS and TNF-α induce VPO1 expression and secretion *in vivo*. We evaluated the VPO1 expression in mouse aorta and plasma after LPS and TNF-α administration. Higher levels of VPO1 in aorta were observed in both LPS and TNF-α treated mice comparing PBS control (Figure 5A and 5B). Plasma VPO1 levels were also elevated after LPS or TNF-α administration (Figure 5C and 5D). These data suggest that VPO1 is able to be induced and accumulated in vessel walls and plasma by pro-inflammatory factors.

### LDL induces VPO1 expression

It is well known that LDL is highly risk factor of atherosclerosis [29, 30]. We then asked if LDL affects VPO1 expression. LDL or oxLDL was administrated to mice. As shown in Figure 5E, LDL induced VPO1 expression in aorta. Very interestingly, oxLDL had higher impact on VPO1 expression than that of native LDL (Figure 5E). Thus, both LDL and oxLDL may induce VPO1 expression in aorta while oxLDL is dominant.

### VPO1-oxidized LDL increases retention in aortic walls

**Figure 6:**
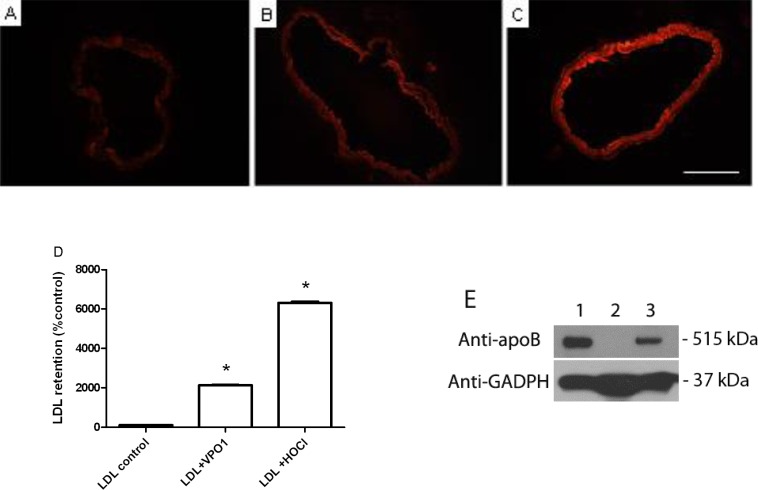
VPO1-oxidized LDL deposits in aortic walls Aortic rings were separated from adult mice (8-12-week old) and incubated in DMEM with 10% FBS as described in Materials and Methods. 40 μg/mL of native LDL (**A.**), VPO1-oxidized LDL (**B.**) or reagent HOCl-oxidized LDL (**C.**) was added into the medium and incubated for overnight. After washing with PBS, aortic rings were stained with DiI for 30 min. Images were recorded by fluorescence microscope. Scale bar, 0.5 mm. Magnification, 20x. (**D.**) Relative fluorescence density of **A.**, **B.** and **C.** Densitometry of DiI staining was analyzed by ImageJ. Fluorescence density of PBS control was as 100%. **E.** Immunoblot analysis of VPO1-oxidized LDL retention in aorta. Mice were injected PBS or oxLDL (52.5 μg). Lane 1, one-year old mouse with oxLDL; Lane 2, two-month old mouse with PBS; Lane 3, two-month old mouse with oxLDL. After 24 hrs, mouse aorta was harvested and subject to immunoblot analysis using anti-apoB polyclone antibody. Anti-GAPDH antibody was as loading control. Data were representative of three mice each group.

We further analyzed the retention of VPO1-oxidized LDL in aortic walls. Aortic rings were separated from adult C57BL/6 mice. The aortic rings were incubated with VPO1-mediated oxLDL or HOCl-mediated oxLDL. As showed in Figure 6A-6C, VPO1-mediated oxLDL significantly increased LDL retention in the aortic rings. This is similar to the positive control, HOCl-mediated oxLDL (Figure 6C). VPO1-mediated oxLDL caused dozen-fold increase of LDL deposition in aortic walls than native LDL (Figure 6D). The result was further confirmed by immunoblot analysis (Figure 6E). The data suggest that VPO1-oxidized LDL is able to deposit in artery walls. Taken together, these data strongly support that VPO1 is potential risk factor of atherogenesis.

## DISCUSSION

Atherosclerosis is a chronic inflammatory disease caused by dysfunction of multiple genes, inflammatory factors and cells. It is generally accepted that hPx enzymes (i.e. MPO) play a critical role in the atherogenesis. However, the exact mechanism of atherogenesis is poorly understood. Previous studies in our laboratory have identified and characterized a novel member of the hPx family, VPO1. VPO1 is unique in the hPx family with its expanding N-terminal domains, widely-spread expression and secretion into blood [16, 17]. To evaluate the role of VPO1 in atherosclerosis, we investigated the LDL oxidation mediated by VPO1 in the presence of Cl- and H_2_O_2_ as well as the effects of VPO1-mediated ox-LDL in genesis of atherosclerosis. VPO1 was demonstrated binding to LDL. VPO1 mediated the oxidation of lipids and apoB-100 of LDL. Exposure of VPO1-mediated oxLDL to human monocyte-like cells led to accumulation of oxLDL and formation of foam cells. Administration of LPS or TNF-α induced VPO1 expression and LDL deposition in artery walls. Our data indicate that VPO1 is a novel and potential risk factor of atherosclerosis.

### The localization of hPx enzymes in aorta and blood

MPO is considered as an important risk factor in atherosclerosis [3, 12, 31]. MPO is strictly expressed in neutrophils and monocytes. Unlike MPO, VPO1 is expressed in multiple cells/tissues [17]. The current study confirms that VPO1 is highly expressed in vessel walls (Figure 5). VPO1 is secreted into the circulation with relatively high concentration (~1.1 μmol/L) [16, 17] while MPO is “leaked” into the plasma (454 to 951 pmol/L) [32]. Plasma VPO1 concentration is approximately 1000 folds than that of MPO. Plasma VPO1 is assumed mainly from vessel walls besed on the data presented in current study and published in other places [16, 17]. Though the VPO1 activity is ~ 5-10% of that of MPO, the total peroxidase activity of VPO1 could be 50-100 folds than that of MPO in plasma. In physiological conditions, there are few MPO proteins in vessel walls while MPO may indirectly enter vessel walls by transcytosis during acute inflammation [13].

### Interaction with LDL

Binding of hPx enzymes to LDL should facilitate the hPx-mediated oxidation of LDL. MPO is highly positively cationic (pI 9.2) and this allows it binding to other molecules and cells such as LDL by electrostatic interaction [33, 34]. The theoretical pI of VPO1 is ~7.0. It seems that VPO1 is less possible to interact with LDL via its charges. However, VPO1 has an expanding N-terminus, which contains five leucine-rich regions and four immunoglobulin C-type domains. These regions and domains are predicted involvement in protein-protein interaction [17]. We have examined the binding ability of VPO1 to LDL. Our data (Figure 1) reveal that VPO1 is able to selectively interact with LDL *via* its leucine-rich regions but not immunoglobulin C-type domains. This interaction may facilitate VPO1-mediated oxidation of LDL, particularly in pathological conditions that VPO1 is over-expressed. The mechanism merits to further investigation.

### The oxidation of LDL and foam cell formation

A line of evidence shows that hPx enzymes play an important role in oxidation of LDL. hPX enzymes mediate LDL oxidation *via* a variety of peroxidation reactions including chlorination, bromination, dityrosine cross-linking and nitration. In these reactions, chlorination is predominant and attributed to MPO [12]. MPO is the member extensively studied [12]. MPO oxidizes both protein and lipid components, increasing their atherogenicity [35] and promotes pathogenesis of atherosclerosis [3]. We previously reported that VPO1 generated HOCl and mediated chlorination of proteins [19]. In the current study, we demonstrate that VPO1 mediates oxidation of apoB-100 and lipids of LDL *via* generating HOCl. With higher expression of VPO1 in vessel walls, our data suggest that VPO1 oxidize LDL locally. Like MPO, ox-LDL mediated by VPO1 also promote formation of foam cells. The production of ox-LDL is an important event in foam cells formation [3, 36], the latter is the hallmark of the pathogenesis of atherosclerosis [3]. Thus, VPO1, like MPO, may contribute to atherogenesis.

### Inflammatory factors and VPO1

At initiation of atherosclerosis, a number of inflammatory factors participate vascular inflammatory responses. These include expression and secretion of chemokines, cytokines, growth factors and enzymes, triggering the activation of a variety of signaling pathways and oxidative stress. Part of inflammatory responses eventually mediates oxidation of LDL and cell damage [37]. For example, LPS administration aggravates atherosclerosis in apoE-deficient mice [26]. Injection of TNF-α into mice caused changes of physiological functions of VECs and VSMCs; those changes, together with others, initiated atherosclerosis [28]. During the initiation of inflammation, increasing cell matrix proteins may be produced by VECs and VSMCs, which facilitate the attachment and oxidization of intimal influx LDL [38]. Thus, the interplay of inflammatory factors and oxidative stress initiates and deteriorates atherosclerosis. LPS and TNF-α were proposed to induce VPO1 expression in vascular endothelial cells [16]. Being consistent with the observations, the current data demonstrate that LPS and TNF-α also highly induce VPO1 expression *in vivo* and secretion into the circulation (Figure 5). Increased VPO1 in vessel walls and blood will potentially mediate the increasing oxidation of LDL. Furthermore, the intimal oxidation and deposition of LDL may cause a series of inflammatory responses characterized by the recruitment of macrophages, phagocytosis of lipoprotein, formation of foam cells, VSMC remodeling, and VEC apoptosis. All of these effects promote formation of atherosclerotic plaque [39]. That VPO1 in aorta is stimulated by LPS or TNF-α sheds light on the studies of hPx enzymes and atherosclerosis. In a future study, we will examine whether there are distinct roles for VPO1 and MPO in pathogenesis of atherosclerosis.

In summary, VPO1, a newly-identified peroxidase, may mediate oxidation of LDL, promote retention of LDL in vessel walls, and form foam cells. VPO1-oxidiazed LDL is readily deposited in aortic walls. Our data suggest that VPO1 may be a novel mediator of atherosclerosis, opening a new avenue to study hPx enzymes and atherosclerosis.

## MATERIALS AND METHODS

### Reagents

NaCl, thiobarbituric acid, sodium hypochlorite solution, 4-Aminobenzoic acid hydrazide (ABAH), lipopolysaccharides (LPS) and catalase were purchased from Sigma-Aldrich (St. Louis, MO, USA); HisPur® Cobalt resin and chemiluminescent substrate for immunoblotting from Pierce Biotechnology (Rockford, IL, USA); rabbit anti-HOCl-oxLDL polyclonal antibody from EMD-Millipore (Billerica, MA, USA); rabbit polyclonal antibody against human apoB-100 from Abcam (Cambridge, MA, USA); tumor necrosis factor (TNF-α) from R&D Systems (Minneapolis, MN); cholesterol kit from Wako Chemicals (Richmond, VA); triglycerides kit from Pointe Scientific (Lincoln Park, MI). Recombinant VPO1 was produced in the laboratory as described in [19]. Rabbit polyclonal anti-VPO1 antibody was affinity-purified [16].

### Animal studies

C57BL/6 mice were used in current study. The protocol was approved by the Institutional Animal Care and Use Committee of the University of Alabama at Birmingham (Animal Project Number: 120309582). Unless otherwise stated, mice were fed with normal chow diet. Mice at 8-12 weeks were used in the experiments.

### Preparation of truncated VPO1

VPO1 29-250 aa and 251-609 aa contain the leucine-rich repeats (LRRs) and immunoglobulin (Ig) C2 domains, respectively. DNAs related to VPO1 29-250 aa and 251-609 aa were subcloned into *E.coli* expression vector, pET30. Truncated VPO1 was over-expressed following the Novagen pET System Manual. Purification was performed using HisPur® Cobalt resin. SDS-PAGE showed that the purity was > 90%.

### Preparation of lipoproteins

LDL was isolated from plasma from healthy volunteers by ultracentrifugation as described in [40]. The protocol was approved by the Institutional Review Board of the University of Alabama at Birmingham. In brief, plasma was layered under 0.9% NaCl (d = 1.006 g/mL), centrifuged for 5 hrs at 100,000×g (4°C). The bottom fraction was collected. The density of the bottom layer was adjusted to 1.063 g/mL with KBr, and centrifuged for 16 hrs at 175,000×g (4°C). LDL was collected by the slicing method. LDL was dialyzed against saline for 48-72 hrs with three changes prior to being used and stored at −80°C.

### Oxidation of LDL

LDL was incubated in 100 μL of phosphate buffer, pH 7.4, containing 500 nmol/L VPO1, 10 μmol/L H_2_O_2_ and 140 mmol/L NaCl for 5 min at 37°C. NaCl alone was as the negative control. The positive control contained 50 nM MPO. Oxidation was initiated by addition of H_2_O_2_ to the reaction. Methionine (5 mmol/L) was added to scavenge HOCl as indicated. In some experiments, ABAH (1 mmol/L) was used to inhibit peroxidase activity.

### Immunoblot analysis

The conventional immunoblot analysis was carried out. In brief, samples were mixed with SDS-PAGE loading buffer [50 mmol/L Tris/HCl (pH 6.7), 2% (w/v) SDS, 200 mmol/L dithiothreitol, 10% (w/v) glycerol, and 0.05% bromophenol blue] and heated in boiling water for 3 min. Proteins were separated by 12.5% SDS-PAGE and transferred to polyvinylidene difluoride membranes (PVDF) membrane. Immunoblots were probed by using the primary antibodies as indicated and visualized by chemiluminescence.

### VPO1-LDL binding experiments

LDL (1 μg or 4 μg) in 100 μl PBS was mixed with 4 μg His-tagged full-length rVPO, rVPO1 (29-250) or rVPO1 (251-609) and incubated for 1 hr at 4°C. 10 μL HisPur® Cobalt resin was then added into the mixture. The mixture was rotated for additional 1 hr at 4°C. The control groups contained His-tagged VPO1 or 4 μg LDL alone. After the incubation, the resin was spun down at 5,000 rpm for 2 min. The supernatant was removed and stored in a new tube. The pellet was washed three times with 1ml of PBS. After final wash, the pellet was resuspended in 30 μL PBS. The pellet and supernatant were subjected to SDS-PAGE and transferred to PVDF membrane. apoB-100 in LDL was detected by using anti-apoB-100 antibody and visualized by chemiluminescence.

### Measurement of free amino groups

Free amino groups of lipoproteins were quantitated as described in [24, 41]. Briefly, 100 μg of LDL was mixed with 1 mL of 4% NaHCO_3_ (w/v; pH 8.4) and 50 μL 0.1% (v/v). trinitrobenzene sulfonic acid. After incubation at 37°C for 1 hr, 100 μL of 1 mmol/L HCl and 100 μL of 10% SDS were added. Absorbance at 340 nm was recorded.

### Measurement of tryptophan residues

Content of tryptophan residues was evaluated as described in [42]. Tryptophan fluorescence was measured at 335 nm using an excitation wavelength of 280 nm with the BioTek Microplate Reader (Winooski, VT). Fluorescence intensity was normalized with protein concentration.

### Electrophoresis of LDL

The electrophoretic mobility of LDL was used for the evaluation of oxidation [43]. LDL or oxLDL was first mixed with Nile red dye, and then was electrophoresed on 1.5% agarose gel at 50 volts for 1.5 hrs. The gel image was recorded and the electrophoretic relative mobility of LDL was measured as the migrating distance of a band divided by the migrating distance of the dye.

### Thiobarbituric acid assay

The thiobarbituric acid assay detects malondialdehyde and malondialdehyde-like derivatives [44]. It was used to assess the extent of LDL lipid oxidation. The thiobarbituric acid-reactive species were quantified at 535 nm using spectrophotometry.

### UV absorbance

UV absorbance of ascorbate during lipid oxidation was performed at 265 nm by using UV-2450 spectrophotometer (Shimadzu Scientific Instruments, Columbia, MD).

### Foam cell formation

Human THP-1 monocytic cells were grown on coverslips in RPMI 1640 medium supplied with 10% fetal bovine serum. To differentiate into macrophages, cells were induced with phorbol-12-myristate-13-acetate (PMA) (100 nmol/L) for 24 hrs. Native LDL or oxLDL (final concentration 100 μg/mL) was added into the culture medium. After 48 hrs, cells were stained by Oil Red O and were observed under microscope. Foam cells as corrected total red oil dye were calculated and quantitated by ImageJ software (The National Institute of Health).

### Induction of VPO1 expression in mouse aorta

LPS (500 ng/g mouse) or TNF-α (8 ng/g mouse) was injected into C57BL/6 mice (8-12-week old) via tail vein. PBS was as control. After 24 hrs, mouse aorta and plasma were harvested. The samples were subject to immunoblot analysis using anti-VPO1 antibody. Densitometry of VPO1 was analyzed by ImageJ. In some experiments, C57BL/6 mice were also injected 52.5 μg of LDL or oxLDL. After 24 hrs, mouse aorta was harvested and subject to immunoblot analysis.

### Induction of apoB-100 expression in mouse aorta

52.5 μg of oxLDL were injected into two-month or one-year old mice (3/group) via tail vein. After 24 hrs, mouse aorta was harvested and subject to immunoblot analysis using anti-apoB polyclone antibody. PBS was as control.

### Aortic ring assay

Aortic rings were prepared from adult C57BL/6 mice (8-12-week old) with the length of ~2 mm. Aortic rings were incubated in DMEM medium containing 10% fetal bovine serum. 40 μg/mL of native LDL, LDL oxidized by VPO1, or LDL oxidized by HOCl was added into the medium. The rings were cultured overnight as described in [45]. After washing with PBS, aortic rings were stained with DiI for 30 minutes. To reduce the non-specific binding of LDL at the ends of aortic rings, the central part of the aortic rings was cut out and used for image analysis. Densitometry of DiI staining was analyzed by ImageJ.

### Statistical analysis

Data were shown as means ± SEM, unless otherwise indicated. Quantitative variables were compared by means of Student's paired t-test for two groups or ANOVA followed by Newman-Student-Keuls test for multiple groups. A value of *P* < 0.05 was considered significant.

## Abbreviations

VPO1: vascular peroxidase −1
MPO: myeloperoxidase
hPx: heme-containing peroxidase
oxLDL: oxidized LDL
VECs: vascular endothelial cells
VSMCs: vascular smooth muscle cells
apoB-100: apolipoprotein B-100
ABAH: 4-Aminobenzoic acid hydrazide
LPS: lipopolysaccharides.
